# Thermal Tightrope: Trajectory Dynamics of Levitating Droplet Clusters under Variable Temperature Conditions

**DOI:** 10.1021/acs.langmuir.6c02750

**Published:** 2026-07-20

**Authors:** Israel A. Huaman, Pavel S. Zun, Alexander A. Fedorets, Michael Nosonovsky, Ekaterina V. Skorb

**Affiliations:** † Infochemistry Scientific Center, 65071 ITMO University, Lomonosov St. 9, St. Petersburg 191002, Russia; ‡ X-BIO Institute, University of Tyumen, Tyumen 625003, Russia; § Department of Mechanical Engineering, 14751 University of Wisconsin-Milwaukee, Milwaukee, Wisconsin 53211, United States

## Abstract

We introduce a comprehensive computational framework for quantifying the dynamics of levitating droplets in a cluster during controlled cooling from 50 to 46 °C. This framework integrates state-of-the-art Computer Vision and Machine Learning techniques such as YOLO-based detection and robust multiobject tracking, to extract kinematic and energetic metrics: estimated velocity, acceleration, kinetic energy, and spatial uniformity. Directional statistics of droplet motion are analyzed through circular statistics, specifically, Mean Resultant Length (MRL) and the Hermans–Rasson (HR) test, to explain temperature-driven changes in trajectory orientation. In addition, we study the efficiency of trajectories defined as the ratio of the straight-line Euclidean displacement to the actual path length. This metric enables objective classification of droplets into high- and low-efficiency groups. Our analysis reveals a dynamical transition at 47 °C. Below this critical temperature, the angular distributions evolve from multimodal to isotropic (uniform/random) behavior, accompanied by a marked reorganization of both MRL and HR values. These findings establish a quantitative link between thermal conditions and collective droplet motion, providing fundamental insights into the stability and self-organization of levitated liquid ensembles.

## Graphical abstract

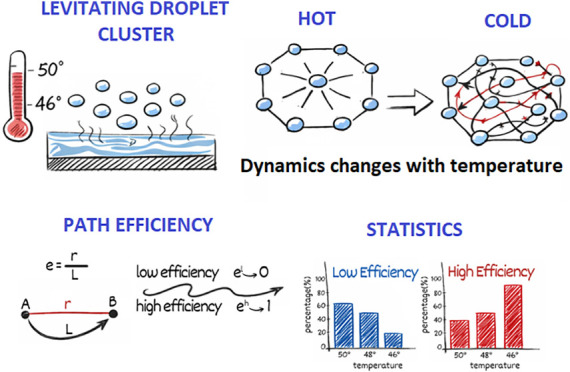

## Introduction

Levitating microdroplets represent an interesting phenomenon at the intersection of fluid dynamics, thermodynamics, and interfacial science, where microscale liquid droplets are suspended above a surface without direct contact. In the case considered in this paper, this levitation occurs when ascending humid air condenses into ordered clusters of microdroplets above a thin layer of water that is heated locally, typically using lasers or other focused heating methods. The droplets are supported by an ascending vapor-air flow generated from the evaporating heated water, creating a cushion that prevents droplets from coalescing with the underlying liquid layer. The droplets may assemble into stable, often hexagonal patterns. This assembly is driven by a balance of aerodynamic forces, evaporative cooling, and interdroplet interactions. The discovery and characterization of such clusters have opened new paths for studying nonequilibrium systems, with their potential applications to microreactors, the study of aerosols in the atmosphere, *in vivo* observation of biological phenomena, and even to computational devices.^1,2^

The physical mechanism underlying this levitation can be traced to the interplay between evaporation-induced gas flows and droplet mutual repelling. When a water surface is locally heated to temperatures between 40–90 °C, intense evaporation produces an upward vapor flow that entrains surrounding humid air. Microdroplets can condense or they can be injected into the system. Such droplets, with a typical diameter of 10–100 μm, levitate at an equilibrium height of several μm to dozens of μm above the surface due to the drag force from the gas flow counteracting gravity. The lateral aerodynamic repulsion results in ordered structures reminiscent of colloid crystal lattices. The Voronoi Entropy (VE) is often used as a measure of orderliness in droplet clusters.

While various static configurations of the levitating droplet clusters, including hexagonal (honeycomb), chain, lacy, hierarchical, and ring clusters have been studied extensively,^3^ dynamic effects in the clusters have not yet been addressed in detail in the literature. Recent studies have explored vibrations and variations in dynamics, such as inverse phase transitions, in which microdroplet clusters exhibit counterintuitive behavior as temperature changes, transitioning from disordered to ordered states.^2^

Note that this phenomenon differs from classical Leidenfrost levitation, which involves larger droplets on superheated solid surfaces, in that it occurs at liquid interfaces and relies on localized heating to control cluster formation and stability.^2^ Subsequent research expanded to include self-stabilization techniques, where salt additions to the water modify evaporation rates to maintain cluster integrity over extended periods.^4^ These advancements have also highlighted the role of environmental factors, such as humidity and dissolved substances, in influencing droplet size distribution and cluster lifespan.^5^ State-of-the-art experimental setups for studying microdroplets now incorporate real-time monitoring to study dynamic behaviors, including oscillations and pattern formation, providing insights into far-from-equilibrium systems that mimic natural processes like cloud droplet organization.^6^

Droplet clusters are also different from traditional colloid crystals formed of rigid particles since the repulsion forces between the droplets are not controlled by the hardcore repulsion. Various aspects of diffusion of droplets within clusters can be studied including such effects as ergodicity breaking, “anomalous diffusion” (subdiffusion and superdiffusion), aging, hydrodynamic memory, molecular crowding, dissipative anomaly, chaos, and fractal trajectories.^7,8^

The efficiency ratio, used in this paper to characterize the trajectories, is a standard measure of trajectory straightness used in the analysis of biological movement,^9,10^ colloidal and soft-matter dynamics.^11^ It has been widely adopted in physics to characterize the quality of directed motion in the presence of noise or obstacle-driven deflections. In our context, the efficiency ratio reflects the competing effects of thermal dynamics (tending to reduce the value) and directed interfacial forces (tending to increase the value).

The integration of machine learning (ML) techniques has revolutionized the analysis and control of levitating droplets, enabling predictive modeling and automated data processing in complex, dynamic environments. ML algorithms are particularly suited for handling the high-dimensional data from droplet clusters, where traditional physics-based models struggle with stochastic elements like turbulent gas flows and variable condensation rates. For instance, neural networks have been employed to track chemical reactions within individual droplets in a cluster.^12^ In levitating systems, ML prediction has proven effective for proactive stability management, as demonstrated by StableLev,^13^ a data-driven pipeline that curates hybrid analytical and experimental data sets of dynamic trajectories to accurately predict and automatically amend instabilities in multiparticle acoustic levitation. Applied to heated water-based clusters, these techniques facilitate the simulation of droplet growth and evaporation, with models like random forests and artificial neural networks forecasting cluster evolution under varying thermal gradients.^14^ Furthermore, ML-driven closed-loop systems have been developed for programmable manipulation, where algorithms process real-time feedback to adjust heating parameters and stabilize clusters for extended experiments.^15^

Complementing ML, computer vision (CV) has emerged as a critical tool for noninvasive characterization of levitating droplets, offering high-resolution spatial and temporal insights into their behavior. Specialized platforms have been designed for microscopical image and video processing tailored to levitating droplet clusters over heated water.^6^ The application of YOLO (You Only Look Once) models to the study of levitating droplets represents a cutting-edge advancement in CV, enabling real-time detection and tracking of dynamic microdroplet clusters in nonequilibrium environments. The DropTrack system^16^ employs YOLOv5 combined with DeepSORT to achieve high-precision droplet detection in complex flows, directly applicable to analyzing the stability and pattern formation in levitating clusters. The synergy between ML and CV, as seen in hybrid frameworks where CV extracts features which ML then uses for classification or prediction,^12^ promises breakthroughs in fields like materials science and environmental engineering.

## Methods

### Experimental Setup

The experiment was conducted using a standard laboratory setup, described in detail in a number of publications.^12,17^ The cluster was formed from a cloud of distilled water microdroplets generated by briefly (≈ 0.5 s) activating an ultrasonic dispenser (Altrasonic, China). An AXIO Zoom V16 stereomicroscope (Zeiss, Germany), equipped with a pco.edge 5.5 camera (PCO AG, Germany), allowed for video recording of the cluster at a frame rate of 50 fps.

The surface temperature of the water beneath the cluster was monitored using a CTL-CF1-C3 pyrometric sensor (Micro-Epsilon, USA; operating spectral range 8–14 μm; NETD 0.1 °C). Condensation growth of the droplets was suppressed by radiation from miniature EK-8520 infrared sources (Helioworks, USA).^18^ This ensured long-term stabilization of the cluster and allowed for the observation of the motion dynamics of droplets within the same cluster across the entire studied temperature range (50 to 46 °C in 1 °C decrements).

### Analysis Algorithms

Following our main pipeling scheme (Figure 1), we present the computational analysis framework, that was implemented as a Python-based application utilizing PyQt5 for the graphical user interface and OpenCV for image processing. The system employs modular architecture with configurable parameters for adaptive analysis of droplet dynamics.

**Figure 1:**
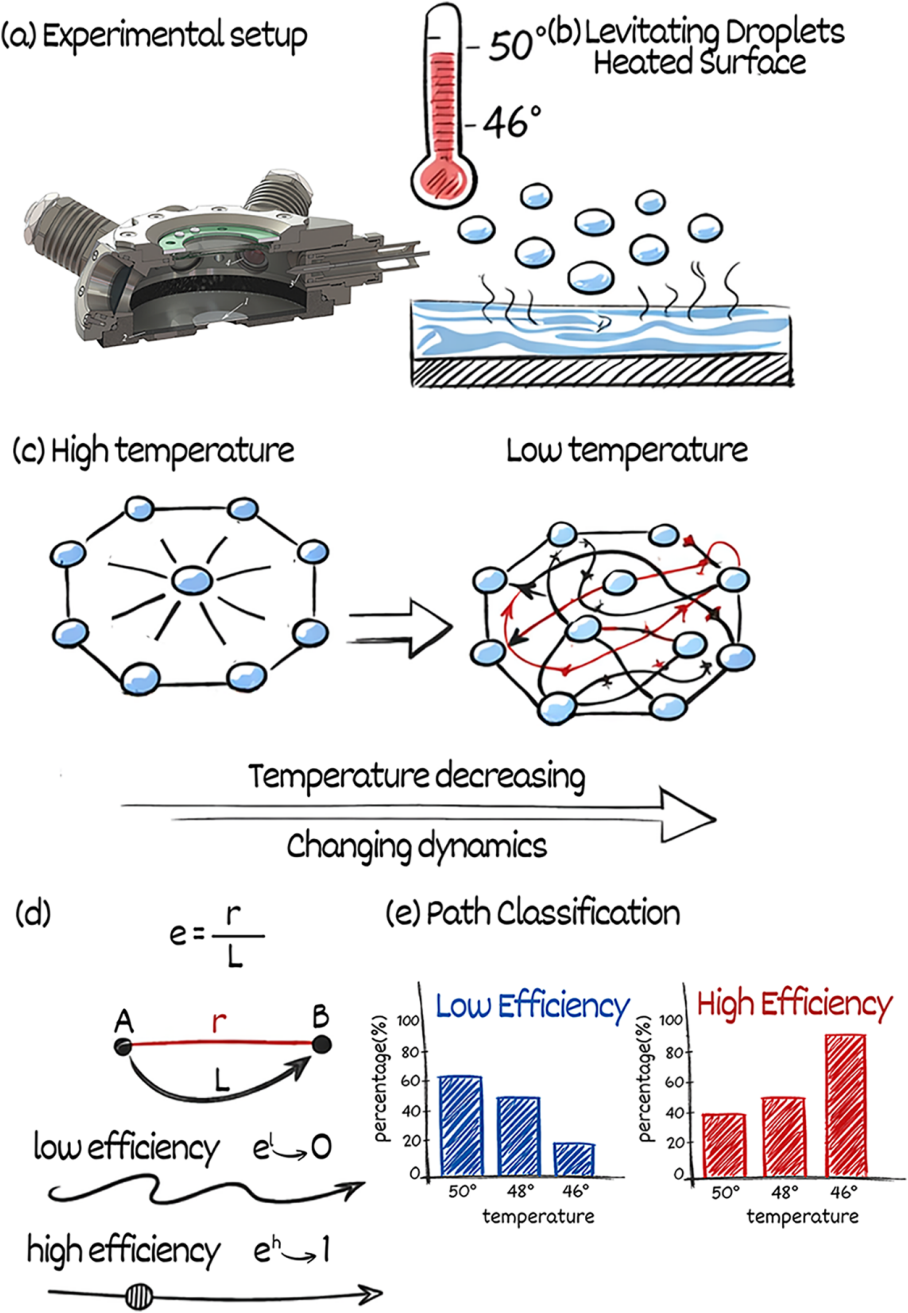
Trajectory analysis for droplets in a cluster. We analyze video recordings of levitating droplets that form a cluster above a heated water surface (a). These videos represent five different experiments with temperatures of the surface ranging from 50 to 46 °C. We study the trajectory dynamics of the droplets, affected by a decremental change of temperature (b). To quantify the changing dynamics (c), we define an *efficiency* value *e* that is calculated as the distance from an initial to a final position *r*, divided by the trajectory length *L*. If the efficiency value is closer to 0 (bottom half of the trajectories), we consider the trajectory as “low efficiency”, otherwise “high efficiency”(d). Finally, we provide insights from the trajectory particularities between temperatures (e).

#### Video Preprocessing and Stabilization

Input video frames undergo stabilization correction to compensate for camera motion and acoustic field vibrations. A moving average filter with configurable window size (default: 5 frames) calculates stabilization offsets based on detected object centroids, ensuring consistent spatial reference throughout the analysis.

#### Object Detection and Tracking

YOLOv11 neural network models perform real-time object detection, identifying droplet positions with bounding box coordinates. The BoT-SORT algorithm maintains object identities across frames, handling occlusions and temporary disappearances with a maximum age parameter (default: 10 frames). The video sequences examined in this work possess characteristics that align favorably with the operational strengths of modern object-detection architectures such as YOLOv11. A uniformly illuminated background, high subject-background contrast, and morphologically distinct droplets that experience limited occlusion or overlap. Under these constrained experimental conditions, the tracker demonstrated robust performance across frames. Tracking data is stored as frame-by-frame position histories for each identified droplet (Figure 2). To address the reliability of the automated tracking, we performed a quantitative uncertainty analysis based on the tracker’s motion model. Specifically, for each video frame, we extracted the trace of the top-left 2 × 2 position covariance matrix (*Pxx* + *Pyy*), which serves as a direct measure of the instantaneous positional uncertainty of each tracked droplet. Our analysis across the five temperature conditions reveals a clear inverse relationship between temperature and tracking uncertainty. The highest confidence (lowest uncertainty) is observed at the highest temperature (50 °C), with a tracker uncertainty of 0.626 ± 0.103. As temperature decreases, the average uncertainty progressively increases, reaching a maximum of 0.354 ± 0.218 at 46 °C. This trend suggests that droplet motion becomes less predictable at lower temperatures, likely due to changes in flow dynamics. These results confirm that while the automated tracker is generally reliable, the confidence in the extracted trajectories is temperature-dependent.

**Figure 2:**
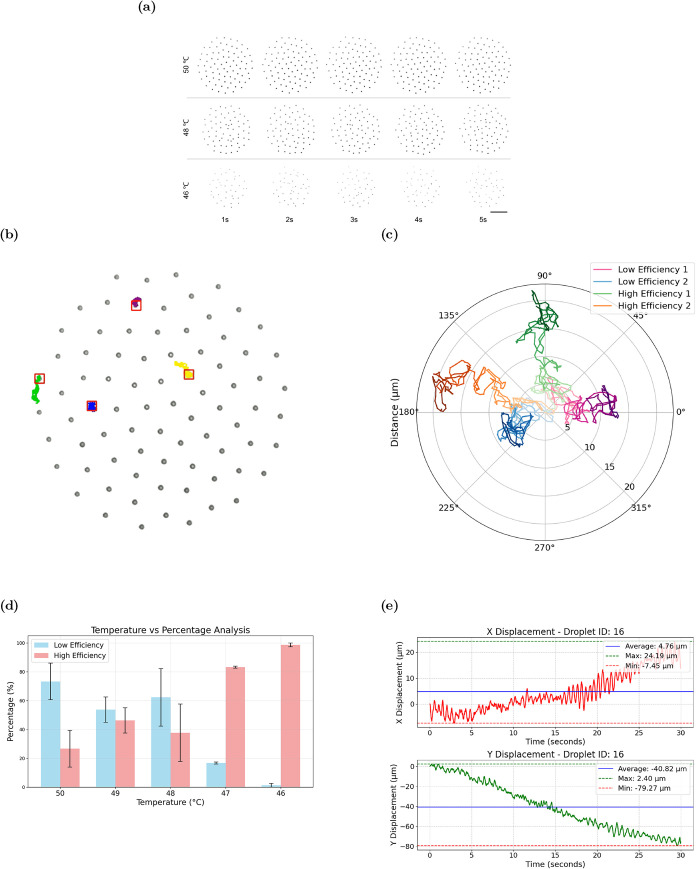
Droplets’ trajectory analysis. Using a high-speed camera, we obtain videos of the microdroplet cluster at 50 frames per second (a) (scalebar: 100 μm). With the help of our trained YOLOv11 model and the BoT-SORT algorithm, we keep track of each droplet’s behavior through time (b). We identify two main types of trajectories according to their efficiency as defined above (c). We perform a group analysis of the droplets with their standard error (d), and we are also able to describe an individual profile of each droplet (e).

#### Metric Calculation

Velocity vectors are computed using finite differences between consecutive positions (with displacement threshold of 1 pixel minimum), with temporal smoothing applied. Acceleration is obtained from second-order derivatives of position data, filtered to eliminate high-frequency noise. Kinetic energy calculations incorporate droplet mass (derived from diameter and density parameters) and velocity measurements. Spatial distribution analysis uses Voronoi tessellation algorithms that partition the levitation space based on droplet centroids, enabling entropy calculations that quantify cluster uniformity. The Shannon entropy of Voronoi cell areas provides a measure of spatial organization, with higher entropy indicating more chaotic distributions.

### Trajectory Efficiency and Classification

#### Definition

Droplet trajectories were quantified using the following efficiency ratio formula:

1 $$e=\frac{r}{L}$$

where *r* is the straight-line Euclidean displacement between the trajectory’s starting and ending positions, and *L* is the total path length (cumulative Euclidean distance along all waypoints). This metric is bounded on the interval [0, 1], with *e* = 1 representing straight-line motion, and *e* → 0 indicating movement in a neighborhood of the starting point.

The efficiency value depends on the trajectory length, and a longer trajectory has the same or (usually) lower values of efficiency than the smaller parts of the same trajectory. To make the efficiency comparable between trajectories, *r* and *L* are measured over the same length of time. This time period must be sufficiently large to avoid noise due to measurement and segmentation errors and per-frame differences; on the other hand, it must be sufficiently small to keep the trajectory length smaller than the cluster size to avoid boundary effects. The efficiency was therefore calculated for time-overlapping chunks of droplet trajectories, each 1 s (50 frames) long. Our preliminary assessment showed the average droplet size of 8–10 μm, the typical velocity of 30–50 μm/s, and the typical cluster size of 250–350 μm; which means the droplets move about 3 to 6 times their diameter per second, and no more than 1/fifth of a cluster size. The 1 s window was chosen to minimize fluctuations while keeping the trajectories significantly below the cluster size. The per-droplet efficiency was then calculated as the average value for all chunks which belong to its trajectory.

#### Trajectory Classification

Based on the efficiency ratio, individual trajectories were partitioned into two dynamically distinct classes (Figure 3):

- *High efficiency*: *e* ≥ 0.5. Trajectories that exhibit minimal deviation from the net displacement direction, indicating either strong directional driving forces or rapid directed motion.
- *Low efficiency*: *e* < 0.5. Trajectories that show significant path elongation relative to end-to-end displacement, with evidence of frequent direction changes or confinement-induced wandering, indicating weaker directional control or higher stochasticity.

The threshold *e* = 0.5 was chosen to achieve an approximate balance in the trajectory population and to create a physically interpretable boundary. Within each class, trajectories were further grouped by temperature (50, 49, 48, 47, 46 °C) yielding ten temperature–class combinations per experiment. For each group, sequences of discrete droplet positions (*X*, *Y* coordinates sampled at 50 fps) were used to compute the angular dynamics using the statistics analysis described in the next section.

**Figure 3:**
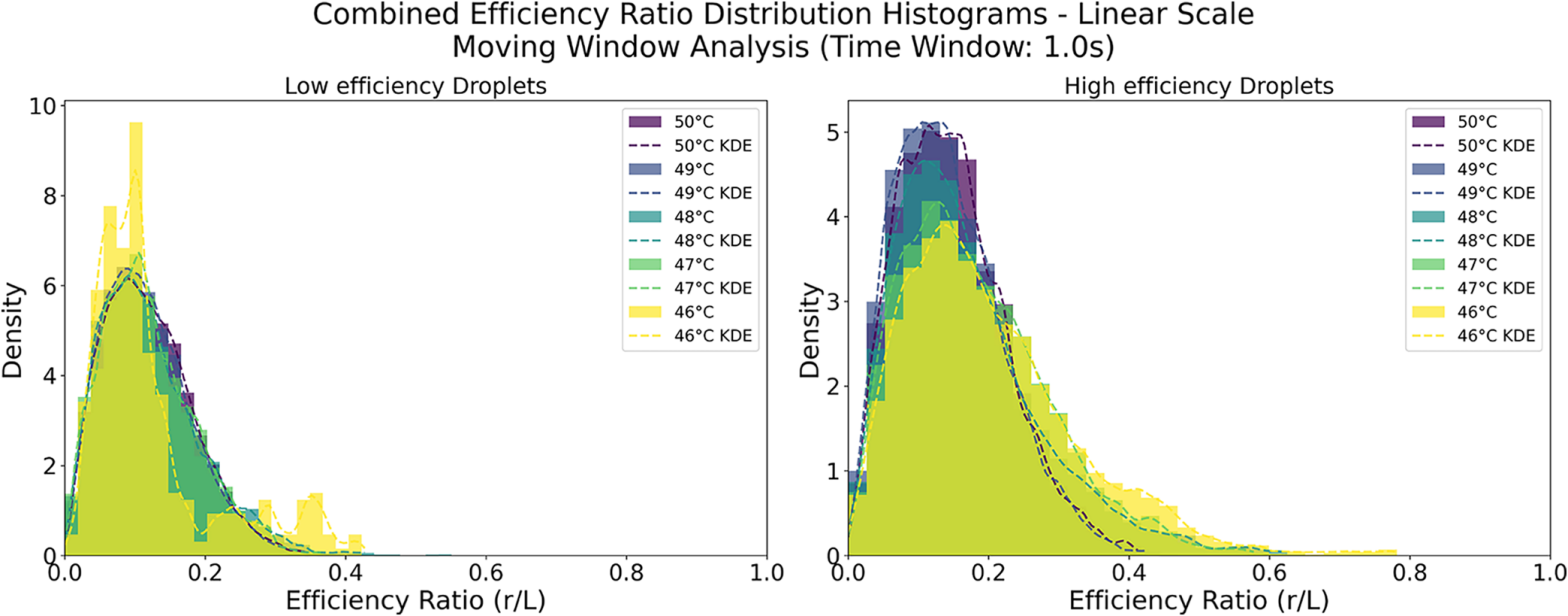
Efficiency distribution per trajectory type. Logarithmic representations of instantaneous efficiency (measured over a 1-s window) of both low (left) and high-efficiency (right) trajectories. For high efficiency the distribution peak is closer to the right and the distribution is wider, suggesting higher variability in the instantaneous values.

The efficiency ratio captures a fundamental aspect of droplet dynamics: in high-temperature regimes, droplets are pushed persistently in one or two dominant directions (high *e*). As temperature decreases, thermal fluctuations and confinement effects dominate, causing trajectories to become more random and exploratory (low *e*).

### Circular Statistics Analysis

#### Mean Resultant Length (MRL)

Directional data (angles) require specialized statistical analysis distinct from linear statistics.^19,20^ For a set of *n* angles {θ_*i*_}_*i* = 1_^*n*^ measured from the droplet trajectories, we first convert to unit vectors:

2 $$\mathrm{x̂}=\frac{1}{n}\sum_{i=1}^{n}\cos\left(\theta_{i}\right),ŷ=\frac{1}{n}\sum_{i=1}^{n}\sin\left(\theta_{i}\right)$$

The resultant vector and its length (MRL) are

3 $$R=\sqrt{{\mathrm{x̂}}^{2}+{ŷ}^{2}}$$

MRL ranges from 0 (uniform/isotropic distribution) to 1 (perfect concentration at a single angle). For bidirectional movement (axial data), angles are doubled before computing MRL:

4 $$R_{\mathrm{axial}}=\frac{1}{n}\sqrt{{\left(\sum_{i=1}^{n}\cos\left(2\theta_{i}\right)\right)}^{2}+{\left(\sum_{i=1}^{n}\sin\left(2\theta_{i}\right)\right)}^{2}}$$

which captures the preference for a given axis regardless of direction along it.

#### Hermans–Rasson Uniformity Test

The Hermans–Rasson (HR) test provides a statistical hypothesis test for directional uniformity.^21^ Given a sample of *n* angles {θ_*i*_}, the original test statistic measures deviation from the uniform distribution on the circle:

5 $$T=\frac{n}{\pi }-\frac{1}{2n}\sum_{i=1}^{n}\sum_{j=1}^{n}\left|\sin\left(\theta_{i}-\theta_{j}\right)\right|$$

Large positive values of *T* provide evidence against uniformity through concentration of angles.

The improved HR statistic, which we used for our calculations,^22^ uses the circular distance δ_*ij*_ = min­(|θ_*i*_ – θ_*j*_|, 2π – |θ_*i*_ – θ_*j*_|) and defines:

6 $$V=\frac{1}{n}\sum_{i=1}^{n}\sum_{j=1}^{n}[\left(\delta_{\mathrm{ij}}-\frac{\pi }{2}\right)-2.805(\left|\sin\left(\theta_{i}-\theta_{j}\right)\right|-\frac{2}{\pi })]$$

The improved version *V* is sensitive to both unimodal concentration (*V* < 0) and multimodal clustering (*V* > 0), making it superior for characterizing arbitrary nonuniform distributions.^22^

To produce statistically meaningful *p*-values despite large sample sizes, we employ a subsampling strategy:^23^ (i) compute the observed test statistic *V*_obs_ on the full data set (all *n* angles); (ii) subsample to n* = 500 angles uniformly at random and recompute the statistic on the subsample *V*_sub_ = *V*(n*); (iii) generate *N*_sim_ = 9999 Monte Carlo simulations under the null hypothesis (uniformly distributed angles); (iv) compute the two-sided *p*-value as

7 $$p=\frac{\#\left\{\left|V_{\mathrm{sim}}-{\mathrm{V̂}}_{\mathrm{sim}}\right|\geq \left|V_{\mathrm{sub}}-{\mathrm{V̂}}_{\mathrm{sim}}\right|\right\}+1}{N_{\mathrm{sim}}+1}$$

where *V̂*_sim_ is the mean test statistic under the null distribution. The two-sided test correctly identifies both concentrated and multimodal departures from uniformity.^21^

## Results and Discussion

The developed analysis framework was applied to levitating droplet cluster experiments across the temperature range of 50 to 46 °C. Individual droplet trajectories were reconstructed with pixel accuracy, enabling detailed characterization of velocity and acceleration profiles. Cluster-level analysis revealed temperature-dependent changes in spatial uniformity, with Voronoi entropy calculations providing quantitative measures of cluster organization. Kinetic energy distributions showed systematic variations with temperature, reflecting changes in droplet interaction dynamics. Trajectory analysis identified distinct behavioral patterns, including stable cluster configurations at higher temperatures transitioning to dynamic reorganization at lower temperatures.

### Circular Statistics for Directional Motion Analysis

We applied the methods described above to quantify directional preferences in droplet trajectories classified by motion efficiency (low vs high) across decremental temperatures ranging from 50 to 46 °C (Figure 4). To obtain meaningful measurements, we averaged the results from three different video samples.

**Figure 4:**
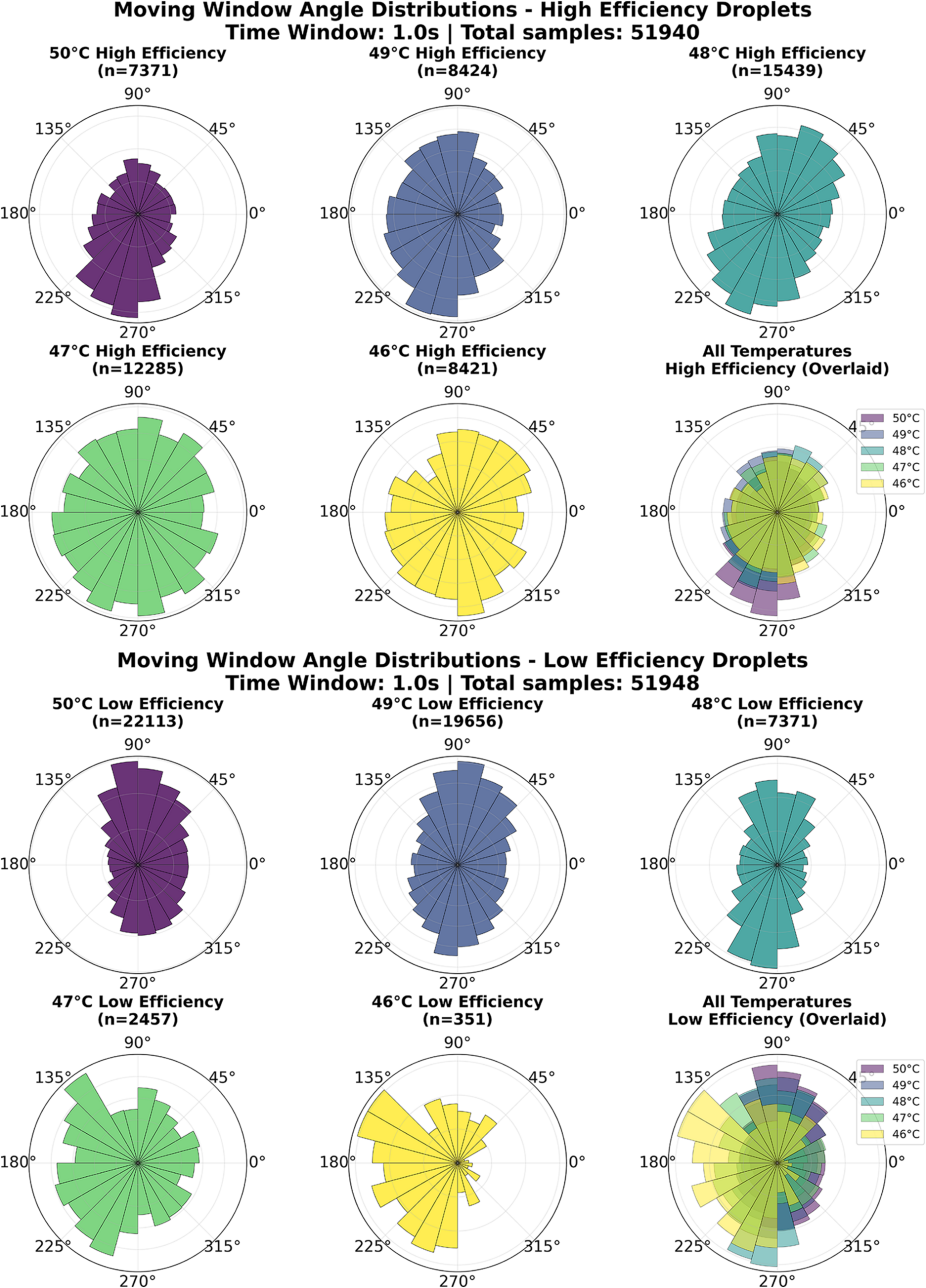
Polar angle distribution of the motion direction averaged over a 1-s moving window per trajectory type for different temperatures. Top: high-efficiency droplet trajectories; bottom: low-efficiency.

#### Mean Resultant Length (MRL) for Bidirectional Assessment

For each temperature–class group, we computed the MRL on doubled angles (eq 4) to assess preference for moving along a given axis regardless of direction. Our results revealed that droplets at 50, 49, and 48 °C exhibited the strongest axial preference (MRL ≥ 0.200), indicating movement was highly oriented along a single axis approximately perpendicular to itself (perpendicular duality at separation ≈ 180°) (Figure 5). We also observed a temperature-dependent weakening, as MRL values decreased from 50 to 46 °C in both classes, with low MRL (<0.13) observed below 48 °C in constrained droplets, indicating transition toward isotropic motion at lower temperatures.

**Figure 5:**
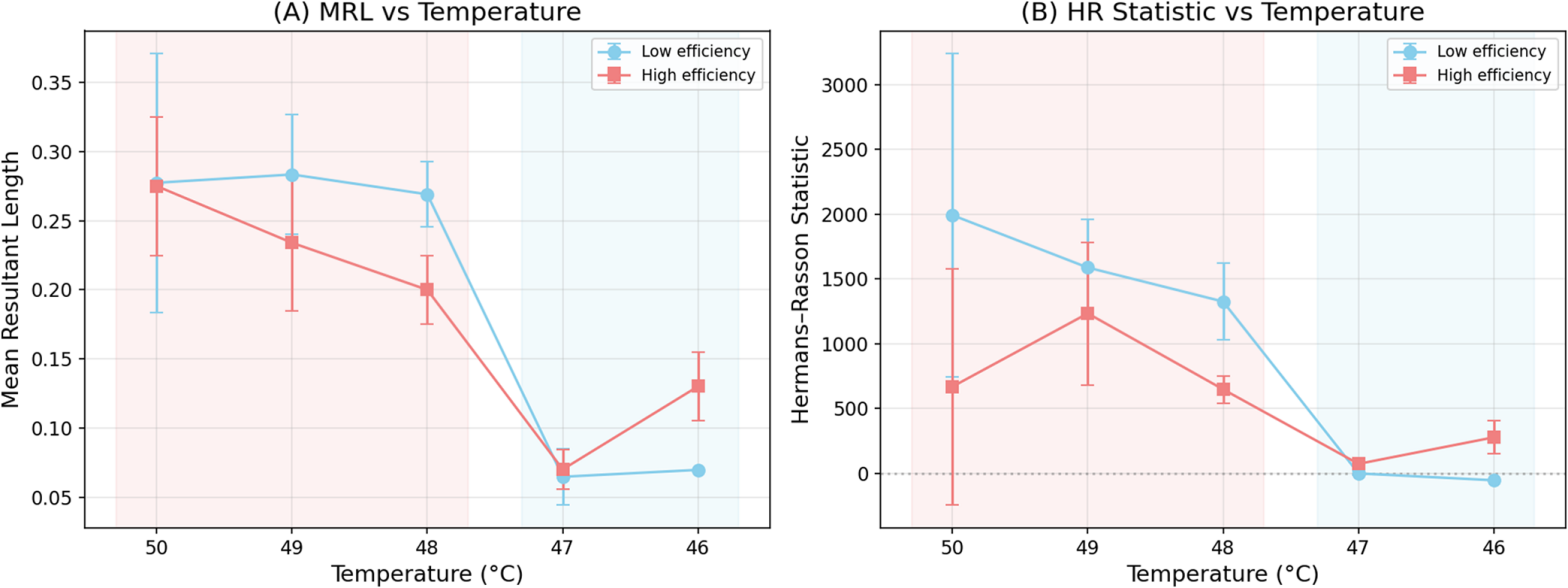
A transition can be seen between 48 and 47 °C. (A) MRL vs temperature and (B) HR vs temperature for low-efficiency and high-efficiency trajectories. The bars correspond to the standard error.

#### Hermans–Rasson Test for Uniformity

The improved HR statistic (*V*) was computed on the full trajectory angles for each temperature–class combination, followed by *p*-value determination through subsampling (n* = 500) and Monte Carlo testing (*N*_sim_ = 9999).

##### Low Efficiency

The HR statistics revealed distinct pattern signatures across temperatures. At 48–50 °C (Bimodal, Significant), large positive HR statistics (*V* = +1325.8 to +1992.7, *p* < 0.001) indicate robust multimodal clustering. The separation between modes (170–180°) approached perfect opposition, suggesting droplets preferentially moved along two distinct directions separated by approximately 180°. At 47 °C (Transition), HR statistics crossed zero (*V* = 0.8, *p* = 0.293), indicating a shift from multimodal clustering toward unimodal concentration; the nonsignificant *p*-value is consistent with this temperature serving as a transition point. At 46 °C (Unimodal, Significant), a negative HR statistic (*V* = −52.2, *p* < 0.001) confirmed statistically significant unimodal concentration near 225°.

##### High Efficiency

At 48–50 °C (Bimodal, Significant), large positive HR statistics (*V* = 648.8 to 1234.3, *p* < 0.001) demonstrated strong bimodal directional preference. At 47 °C (Transition), very low HR statistic (*V* = +74.4) suggested a change of tendency, but Monte Carlo testing yielded *p* = 0.087 (not significant), representing a critical transition zone. At 46 °C (Uniform, Not Significant), small positive HR statistics (*V* = +278.9, *p* = 0.007) with medium MRL (0.130) indicated isotropic motion consistent with near uniformly distributed directions.

## Discussion

Previous studies of the droplet cluster concentrated mostly on the self-assembled cluster with static configurations, such as hexagonal and chain clusters. The dynamic behavior of droplets in clusters is much more diverse, and it can be investigated with the methods presented in this article.

One particular question is the behavior of the trajectories, which are, on the one hand, subject to random forces acting from the gas flow, but on the other hand, their motion is severely constrained by being a part of the cluster and interacting with other droplets. This behavior may demonstrate some features of diffusion and some features of convective motion.

For the classical (Einstein–Smoluchowski) diffusion, the root-mean-square displacement is proportional to the square root of the lag time. Contrary to that, for anomalous diffusion the dependency has the form of a power law with an exponent α < 0.5 for subdiffusion. There are many reasons why particle trajectories demonstrate subdiffusion behavior: macromolecular crowding, flowing through obstacles, the so-called “hydrodynamic memory” caused by vortices, etc. Consequently, the friction force depends on the entire history of the particle’s trajectory, and it is related to the fractal nature of a turbulent trajectory.^8^

The results presented in this paper show the presence of a qualitative transition in droplet dynamics for levitating clusters at a particular temperature, and present ML and statistical tools to quantify this transition.

The authors previously studied small oscillations of a microdroplet cluster levitating in an ascending vapor stream and found that the cluster tends to oscillate as a whole.^24^ They showed that the synchronization of droplets’ trajectories is not caused by the interactions between droplets, but by external fluctuations with the characteristic length scale much larger than the size of the droplets. The center of mass of the entire cluster produces the same motion as single microdroplets, indicating ergodic behavior. In all these cases, the fractal behavior is responsible for ergodicity breaking and for transport deceleration in comparison with the classical diffusion law.

## Conclusions

The combined evidence from MRL, HR statistic, *p*-values, and mode separation converges on the same conclusion: droplet displacement at high temperatures is governed by structured, directional dynamics (strong bimodal/unimodal angular distributions), while at low temperatures the angular distributions transition toward isotropy, indicating a fundamentally different dynamical regime.

## Supplementary Material



## Notes

Tables S1–S3 containing descriptive analyses of low- and high-efficiency trajectories and tracker uncertainty across evaluated temperatures (PDF)
