# Supplementary material for: Thermal Tightrope: Trajectory Dynamics of Levitating Droplet Clusters under Variable Temperature Conditions
Source: Langmuir. 2026 Jul 20;42(31):22929–37. doi: 10.1021/acs.langmuir.6c02750 (PMC13472157; doi:10.1021/acs.langmuir.6c02750)
Supplement: Supplementary file 1 [file la6c02750_si_001.pdf]

Supporting Information

On the Thermal Tightrope: Trajectory Dynamics of Levitating Droplet Clusters Under Variable Temperature Conditions

Israel A. Huaman[1], Pavel S. Zun[1,2], Alexander A. Fedorets[2], Michael Nosonovsky[1,3*], Ekaterina V. Skorb[1]

[1]Infochemistry Scientific Center, ITMO University, Lomonosov St. 9, St. Petersburg, 191002, Russia

[2]X-BIO Institute, University of Tyumen, Tyumen, 625003, Russia

[3]Department of Mechanical Engineering, University of Wisconsin-Milwaukee, Milwaukee, WI 53211, USA

***Corresponding author:** nosonovs@uwm.edu

Table of Contents

- Table S1. Descriptive analysis of low-efficiency trajectories.
- Table S2. Descriptive analysis of high-efficiency trajectories.
- Table S3. Tracker uncertainty (mean positional covariance trace ± standard deviation) for each evaluated temperature condition.

Table S1: Descriptive analysis of low-efficiency trajectories.

Temperature (°C)	MRL	HR stat	p-val
50	0.277	+1992.7	< 0.001
49	0.283	+1588.1	< 0.001
48	0.269	+1325.8	< 0.001
47	0.065	+0.8	0.293
46	0.070	−52.2	< 0.001

Table S2: Descriptive analysis of high-efficiency trajectories.

Temperature (°C)	MRL	HR stat	p-val
50	0.275	+668.7	< 0.001
49	0.234	+1234.3	0.001
48	0.200	+648.8	< 0.001
47	0.070	+74.4	0.087
46	0.130	+278.9	0.007

Table S3: Tracker uncertainty (mean positional covariance trace ± standard deviation) for each evaluated temperature condition.

Temperature (°C)	Tracker uncertainty
50	0.626 ± 0.103
49	0.558 ± 0.121
48	0.486 ± 0.141
47	0.421 ± 0.192
46	0.354 ± 0.218
